# A major QTL at the *LHCGR/FSHR* locus for multiple birth in Holstein cattle

**DOI:** 10.1186/s12711-021-00650-1

**Published:** 2021-07-03

**Authors:** Sarah Widmer, Franz R. Seefried, Peter von Rohr, Irene M. Häfliger, Mirjam Spengeler, Cord Drögemüller

**Affiliations:** 1 Institute of Genetics, Vetsuisse Faculty, University of Bern, 3012 Bern, Switzerland; 2 Qualitas AG, 6300 Zug, Switzerland

## Abstract

**Background:**

Twin and multiple births are rare in cattle and have a negative impact on the performance and health of cows and calves. Therefore, selection against multiple birth would be desirable in dairy cattle breeds such as Holstein. We applied different methods to decipher the genetic architecture of this trait using de-regressed breeding values for maternal multiple birth of ~ 2500 Holstein individuals to perform genome-wide association analyses using ~ 600 K imputed single nucleotide polymorphisms (SNPs).

**Results:**

In the population studied, we found no significant genetic trend over time of the estimated breeding values for multiple birth, which indicates that this trait has not been selected for in the past. In addition to several suggestive non-significant quantitative trait loci (QTL) on different chromosomes, we identified a major QTL on chromosome 11 for maternal multiple birth that explains ~ 16% of the total genetic variance. Using a haplotype-based approach, this QTL was fine-mapped to a 70-kb window on chromosome 11 between 31.00 and 31.07 Mb that harbors two functional candidate genes (*LHCGR* and *FSHR*). Analysis of whole-genome sequence data by linkage-disequilibrium estimation revealed a regulatory variant in the 5ʹ-region of *LHCGR* as a possible candidate causal variant for the identified major QTL. Furthermore, the identified haplotype showed significant effects on stillbirth and days to first service.

**Conclusions:**

QTL detection and subsequent identification of causal variants in livestock species remain challenging in spite of the availability of large-scale genotype and phenotype data. Here, we report for the first time a major QTL for multiple birth in Holstein cattle and provide evidence for a linked variant in the non-coding region of a functional candidate gene. This discovery, which is a first step towards the understanding of the genetic architecture of this polygenic trait, opens the path for future selection against this undesirable trait, and thus contributes to increased animal health and welfare.

## Background

In dairy cattle production, herd profitability is heavily influenced by the number of calves born alive and by the length of calving intervals. Intensive selection for milk yield in dairy cattle has led to a decline in female fertility, due to unfavorable genetic correlations between milk yield and female fertility [1, 2]. Improved management practices and genomic selection have contributed to reversing negative trends in dairy cow fertility, but further progress is still required.

Genome-wide association studies (GWAS) have become a useful tool to partially reveal the genetic architecture of complex traits. For several traits related to female fertility, such as non-return rate at 56 days and interval from first to last insemination, quantitative trait loci (QTL) have been detected e.g. in Holstein [3] and Brown Swiss cattle [4]. Mining of population-based whole-genome sequence (WGS) datasets in the associated genomic regions has been used to identify causative variants for both monogenic Mendelian and polygenic complex traits [5]. The necessary tools, such as GWAS and linkage disequilibrium mapping, are available for the analysis of other female fertility traits such as multiple birth.

Cattle are generally a monotocous species and pregnancies typically result in the birth of singletons. Multiple births are rare, with multiple birth rates (MBR) ranging from 1.02 to 9.6% depending on the breed and study [6–15] and are generally higher in dairy cattle than in beef cattle [6]. Most multiple births result from multiple ovulations when two or more ovulatory follicles mature simultaneously. In cattle, only about 5 to 10% of the twins are monozygotic. Silva del Rio et al. [16] found that 7.5% of the twins were monozygotic and Atteneder [6] determined a rate that ranges from 6.5 to 11.7% depending on the breed analysed. However, to date, this trait has not been analyzed in general or with a genetic model using data from Switzerland.

For dairy cattle, twins and multiple births are undesirable for several reasons. Multiple births are generally associated with increased health problems for both the dam and the calves. Occurrence of remained placenta, metabolic disorders, displaced abomasum, and ketosis are some of the direct negative effects on the dam [17–21]. The impact of multiple births on fertility is indicated by its effect on the subsequent calving interval and conception rate [18, 22–24]. Calves from multiple births also have a higher risk of mortality and deficiency syndrome; the risk of abortion, dystocia and stillbirth also increases with multiple births [6, 17, 18, 20, 22–26]. All these factors result in higher costs for the farmers. Thus, selection against this trait could improve fertility and as well as profitability of dairy operations.

There are different non-genetic factors which might influence MBR. Several studies have shown that the parity of the cow influences MBR significantly, as well as other environmental factors such as season and herd [6–11, 26, 27]. Cows in parity one (0.70 to 1.63%) have significantly lower MBR than multiparous cows (2.87 to 7.35%), and MBR was found to increase until the 3rd to 5th parity and then to remain stable in later lactations. Two studies have analyzed the association between milk yield and MBR. While one study found no association [12], the second suggested an association of higher MBR with higher milk yield [13]. Calving season seems to be an important factor that affects the variation in MBR, with different studies reporting a higher MBR for births occurring in the summer months [6–10, 26]. These results suggested that the rate of multiple ovulations at conceptus is higher in late summer and fall, although another study reported that MBR is highest between the end of summer and fall [27]. A positive phenotypic trend for MBR over time has been observed in several studies [8, 10, 11, 14, 26–28], which indicates that MBR is associated with other traits under selection. Reports on the genetic trend for MBR are conflicting, with one study showing a negative trend over time [7] and another one finding no association between MBR and time [13]. Overall, MBR is increasing in most of the dairy cattle populations analyzed to date. Atteneder [6] suggested that the age at first calving affects MBR, with older dams having a higher rate. A heritability of 0.011 to 0.160 was estimated for MBR, which indicates a low but non-zero genetic contribution [7, 9, 10, 12, 14, 15, 27, 29, 30]. Estimations from linear models were lower than those from threshold models. Furthermore, Atteneder [6] showed that the maternal heritability for MBR was higher than the direct heritability (0.017 to 0.063 vs. 0.001 to 0.005) [6]. Hence, it is likely that multiple loci are influencing the trait, which have not been all identified.

Different QTL mapping studies have analyzed maternal multiple birth in North American Holstein, Israelian Holstein or Norwegian cattle using family-based microsatellite interval mapping approaches [15, 31–38] and have identified several QTL on 13 chromosomes, depending on the population examined and the study [15, 36–38]. For North American Holsteins, paternal half-sib families were analyzed using single-marker analysis or combined linkage-linkage disequilibrium approaches resulting in the detection of QTL on eight chromosomes [31, 34]. One study analyzed the Maremmana beef breed in Italy by using a single-trait linear mixed effect model and an animal threshold model that included the number of calves born per cow as the phenotype [14], and detected one significant SNP on chromosome 24 using a GWAS based on 54 k SNP data. The analyses, which we present here based on large-scale phenotype and genotype data and linear mixed models to analyze multiple birth in cattle, are the first in the field.

In this study, our aim was to conduct a comprehensive genetic analysis of the trait multiple birth in Swiss Holstein cattle. To estimate breeding values, we used phenotypic data for single and multiple birth cases that have been recorded over several decades through the national animal recording database and combined them with pedigree data. In addition, we used large-scale genotype data that were obtained during routine genomic selection of males and females to identify associated QTL by GWAS and haplotype regression analysis. A fine-mapping approach was carried out to define a critical genomic region and potential candidate causal variants. Finally, the detected association was validated by evaluating the effect of the identified haplotype on available birth and fertility traits.

## Methods

### Phenotypes

Large-scale phenotypic recording of birth records was carried out routinely through the Swiss national animal recording database between 2006 and 2018. Here, we focused on data from only one breeding organization: swissherdbook (Zollikofen, Switzerland). The raw dataset contained 3,977,467 birth records mainly from the Holstein and Simmental breeds. Data analysis and preparation for breeding value estimation were performed with an inhouse software written in R [39] using RStudio [40]. Birth records resulting from embryo transfer were removed. After data validation and preparation, 971,613 records (235,053 on Holstein, 190,243 on Simmental, 536,932 on Swiss Fleckvieh (Holstein × Simmental), 6726 on Monbéliarde, 1511 on Normande, 1088 on Pinzgauer, and 60 on Evolène) including a multiple birth code were available for the genetic analyses of the discrete trait multiple birth. The overall MBR was 3.56%. Further details on the final dataset are in Table 1.

**Table 1:** Solutions for fixed effects of the estimated breeding values based on the final dataset

Fixed factor	Level	Number of observations per level^a^	Solution for effect
Parity	1	229,669	0.305
	2	229,034	0.356
	3	169,509	0.368
	4	123,887	0.374
	5+	219,514	0.379
Sexed semen	No	920,157	0.354
	Yes	51,456	0.346
Season of birth	Spring	186,693	0.346
	Summer	185,133	0.364
	Fall	313,158	0.354
	Winter	286,629	0.351

^a^Based on the final dataset of 971,613 records

### Estimation of variance components

A mixed linear model was fitted to the phenotypic data described as follows:

1 $${\mathbf{y}} = {\mathbf{Xb}} + {\mathbf{Wh}} + {\mathbf{Z}}_{{\mathbf{d}}} {\mathbf{mb}}_{{\mathbf{d}}} + {\mathbf{Z}}_{{\mathbf{m}}} {\mathbf{mb}}_{{\mathbf{m}}} + {\varvec{\upepsilon}},$$

where $\mathbf{b}$ represents the vector of the fixed effects, $\mathbf{h}$ is the vector of the random herd-year effect, ${\mathbf{m}\mathbf{b}}_{\mathbf{d}}$ and ${\mathbf{m}\mathbf{b}}_{\mathbf{m}}$ represent the direct (calf) and the maternal (dam) genetic effects, respectively, and ${\varvec{\upepsilon}}$ represents the residual. The fixed effects for parity, season, semen sexing, and the covariate of age of dam at birth were considered. The number of records per level of fixed effect are shown in Table 1. $\mathbf{X}$, $\mathbf{W}$, ${\mathbf{Z}}_{\mathbf{d}}$ and ${\mathbf{Z}}_{\mathbf{m}}$ are the design matrices for the fixed ($\mathbf{X}$) and random ($\mathbf{W}$, ${\mathbf{Z}}_{\mathbf{d}}$, and ${\mathbf{Z}}_{\mathbf{m}}$) effects. Model (1) is based on a previous unpublished study that analyzed a similar dataset. The components in the vector of observations ($\mathbf{y}$) were encoded with 1 as single birth, 2 for twin or triplet births. The dataset was filtered to exclude from the analysis all the records from herds that had less than 260 records per herd, and all records in herd-year classes that had less than five records per herd-year class, which resulted in a final dataset of 167,703 records. Variance components were estimated using the software-program vce [41].

### Prediction of breeding values

Breeding values were predicted with the MiX99 program [42] using the mixed linear effects model (1) shown above. The dataset was filtered to exclude all records from herds that had less than 260 records but without setting a minimal number of observations per herd-year levels. In total, 971,613 records were used for breeding value prediction. The required variances and covariances were based on the values estimated in the previous step. Reliabilities of breeding values were estimated based on the approach by Tier and Meyer [43].

Breeding values were standardized to have a mean of 100 and a standard deviation of 12. We defined base animals, the 8- to 10-year old Holstein sires (Red Holstein and Holstein), which is similar to the definition of base animals for the calving ease trait used in Switzerland.

Estimated breeding values (EBV) for the direct (mbd) and maternal (mbm) multiple birth traits were de-regressed according to Garrick et al. [44] (Table 2). De-regressed EBV were used in the association analyses if the corresponding EBV reliability was ≥ 0.35, which left 728 and 2540 animals for the mbd and mbm traits, respectively.

**Table 2:** Statistics of the de-regressed breeding values (BV) for the direct (mbd) and maternal (mbm) multiple birth traits

Trait	Min de-regressed BV	Max de-regressed BV	Mean de-regressed BV (sd)	Number of observations
mbd	− 359.479	119.458	3.453 (35.320)	881
mbm	− 117.343	307.131	2.852 (40.060)	3220

### Genotypes

Routine SNP genotype data generated for genomic selection were available for ~ 60,000 animals. Animals were genotyped using several routinely available array chips that include between 3 and 150 k SNPs. The available genotype archive was used in a two-step imputation approach and was imputed first to a density of 150 k. Subsequently, imputation to HD-density was carried out using 150 k data. The reference dataset for the 150 k array included 1688 Holstein and 1511 Simmental animals and the reference dataset for the HD-density array included 703 Holstein and 663 Simmental cattle. FImpute software was used with default parameters for both steps [45]. In each step, SNPs with a minor allele frequency (MAF) lower than 1% were removed from the dataset. The final marker set included 114,657 and 691,222 SNPs for each density (150 k and HD), respectively. SNPs were filtered using the following thresholds: MAF higher than 0.01 and an SNP call rate higher than 0.99 in the genotype data from the reference population. Filtering was done separately for the Simmental and Holstein populations. The marker set used in both imputation steps was created by selecting SNPs that met the criteria in at least one of the two breeds, Simmental and Holstein. The current ASR-UCD1.2 cow assembly was used as the reference genome during imputation. The mean distance between SNPs in the final HD-density dataset with 691,222 SNPs was 3595 bp.

### Association studies

#### Single SNP regression

Genome-wide single marker association studies were carried out using the mixed model approach and the software snp1101 [46]. Only data from animals with a Holstein pedigree-based gene proportion higher than 0.6 were included in all analyses. After calculating the genomic relationship for the animals used in the single-marker association analyses, it was fitted in the model to correct for population stratification [47], as follows:

2 $$y_{i} = \mu + \beta g_{i} + a_{i} + \varepsilon _{i}$$

where ${y}_{i}$ is the de-regressed EBV of animal $i$, $\mu$ is the overall mean, $\beta$ is the allele substitution effect, ${g}_{i}$ is the SNP genotype of animal $i$, which was coded as 0, 1, and 2 for SNP genotypes *AA*, *AB*, and *BB*, respectively. ${a}_{i}$ is the random additive polygenic effect of animal $i$ with ${\mathbf{a}}\,\sim \,N\left( {\mathbf{0},{\mathbf{G}}\sigma _{a}^{2} {\text{~}}} \right)$ where $\mathbf{G}$ is the genomic relationship matrix [47] and ${\sigma }_{a}^{2}$ is the polygenic additive genetic variance. ${\varepsilon }_{i}$ is the random residual effect. We used this model to identify variants that were significantly associated with the traits mbd and mbm.

#### BayesB approach

Fitting one genotype at a time can easily lead to biased results due to stratification and linkage disequilibrium (LD) [48]. Fitting subsets of genotypes at many markers simultaneously can address this paradigm. Window-based association analyses were conducted using the GenSel software package [49] and the BayesB algorithm [50]. The $\pi$ parameter that represents the proportion of loci with zero effect was estimated beforehand using the same dataset, and a value of 0.989 was set as an a priori starting value. Genomic windows were constructed for 1-Mb segments and the window variance was estimated.

#### Haplotype analysis

A subsequent fine-mapping approach was used to detect possible causal variants. To identify the haplotype that encompasses the putative causal QTL, we performed a haplotype regression analysis limited to the previously identified genomic windows that explained a significant proportion of the total genetic variance. Following an approach described by Pausch et al. [51], our aim was to identify significantly associated haplotypes within each window. Therefore, we estimated haplotype effects by cutting the haplotypes using different lengths of all odd numbers between 9 and 301 SNPs within each identified segment. By shifting the starting point SNP-wise and using different haplotype lengths, each haplotype within each segment was considered in the analysis. Again, de-regressed EBV were used in the haplotype association analysis as response variables. The most significantly associated haplotype was used in the following steps to identify any candidate genomic variants in the whole-genome sequence data. The observed top-associated haplotype for significantly associated windows was tested for association with routinely recorded fertility and birth traits using the GCTA software package [52] and by fitting the haplotype as a single diplotype. The genomic relationship was included in the model to correct for population stratification. In addition, association analyses using de-regressed EBV for routinely available fertility and calving traits were carried out, to test the likely co-association between the most significantly associated haplotypes for twinning and other fertility traits.

### Fine-mapping

Using the plink software package [53] and decoded diplotype data derived from the diplotype of the most significantly associated haplotype, WGS data were screened for variants that were in significant LD with the haplotype. A 880-kb window between positions 30,474,126 bp and 31,350,487 bp on chromosome 11 was chosen based on the localization of the highly associated haplotypes. We used WGS data on 4109 animals provided by the 1000 Bull Genomes Project run 8 that includes 1121 Holstein cattle [54]. The data were converted chromosome-wise from VCF to plink input files using the software VCFtools v0.1.16 [55]. Furthermore, the data were converted to binary files and the VCF quality controls were performed with the plink v.1.90b3.46 software [53]. Variants with a missing call rate higher than 0.1, a MAF lower than 0.01, and samples with missing call rate higher than 0.1 were removed. Mendelian errors were analyzed and all samples and variants that had a Mendelian error rate higher 2% were removed. Pedigree records for the 1000 Bull Genomes Project samples were obtained from the swissherdbook database (Zollikofen, Switzerland) and comprised 544 duos and 10 trios for Mendelian analyses. For the LD-based variant search approach, 318 samples of the 1121 whole-genome sequenced Holstein animals for which additional SNP genotype information was available, were used after data filtering and included 4323 variants located in the 880-kb window on chromosome 11.

To obtain supporting evidence for a putative causal role of the variants, an analysis of sequence homology among 20 mammals was performed with the UCSC genome browser. All variants that were in high LD (r^2^ ≥ 0.7) with the identified associated haplotype were analyzed.

## Results

### Estimation of variance components

The raw values of the estimated variance components are in Table 3. The heritability of the direct and the maternal genetic effects were 0.0015 and 0.0348, respectively.

**Table 3:** Estimated raw variance components

Component	Raw value	Standard error
Herd-year	0.992 E−04	0.185 E−04
Animal	0.578 E−04	0.354 E−04
Dam	0.130 E−02	0.134 E−03
Residual	0.036	0.136 E−03
Correlation animal/dam	− 0.158 E−03	0.816 E−04

Based on the used dataset of 167,703 records

### Prediction of breeding values

**Fig. 1:**
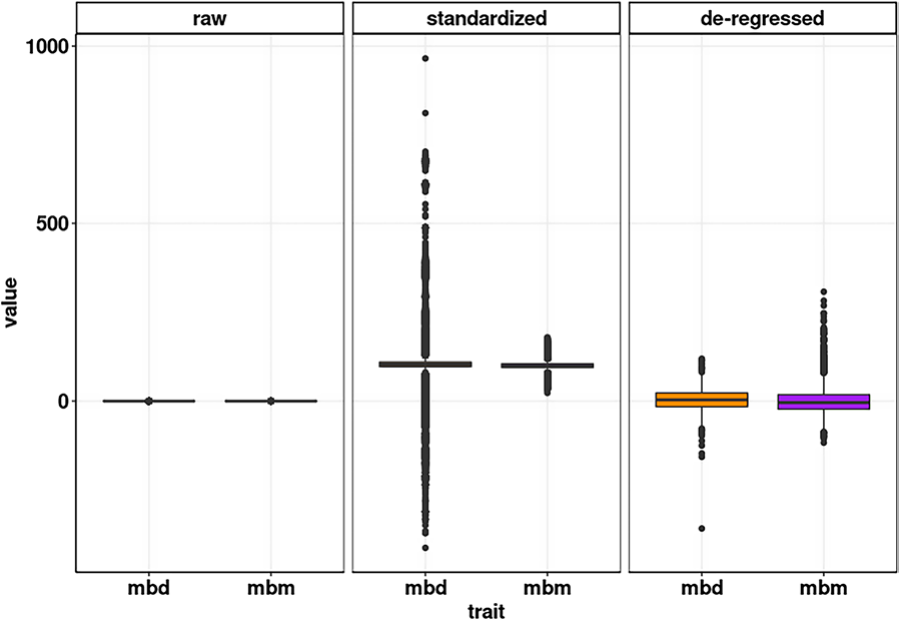
Raw, standardized, and de-regressed breeding values for the direct (mbd) and maternal (mbm) multiple birth traits. The standard deviation (SD) for the standardized breeding values is 35.959 for mbd and 8.691 for mbm

Breeding values were estimated for the traits mbm and mbd, and these were used in a de-regressed transformation as input for the association analysis. The solutions for the fixed effect of parity, use of sexed semen, and season of birth are in Table 1. We found a significant negative effect of the use of sexed semen on multiple birth rate. Furthermore, the highest prevalence for multiple births was observed in summer, and multiparous cows had a higher incidence for multiple births. The empirical distributions of the raw, standardized, and de-regressed estimated breeding values are shown as boxplots in Fig. 1.

**Fig. 2:**
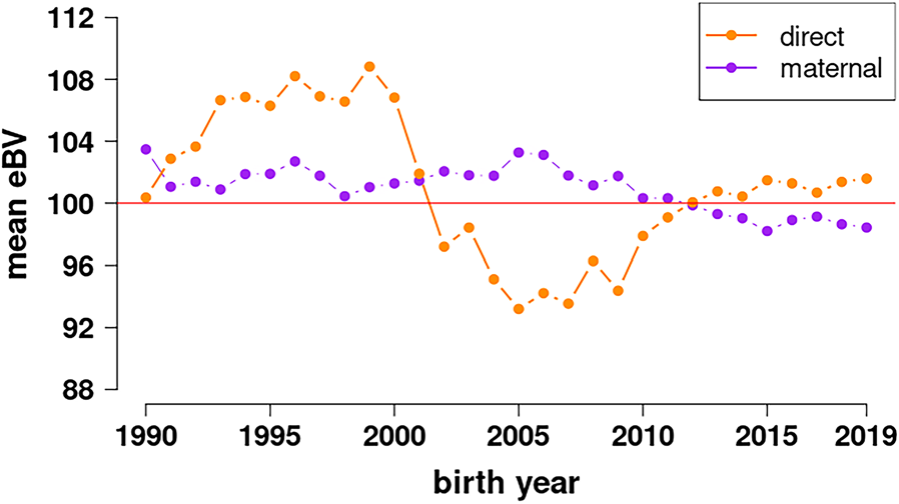
Genetic trend of the estimated breeding values of multiple birth from 1990 to 2019

The mean reliability for the standardized breeding values of 1,750,016 animals was 0.144 for mbd and 0.219 for mbm. The mean estimated breeding values grouped by year of birth for all the animals is a measure of the genetic trend of a trait. Interestingly, no clear genetic trend (Fig. 2) was observed for either of the traits analyzed, mbd and mbm, which led us to speculate that they have not changed during the last decades, neither directly due to selection pressure nor indirectly due to any correlated selection response.

### Association studies reveal a major QTL on chromosome 11

#### Single SNP regression

**Fig. 3:**
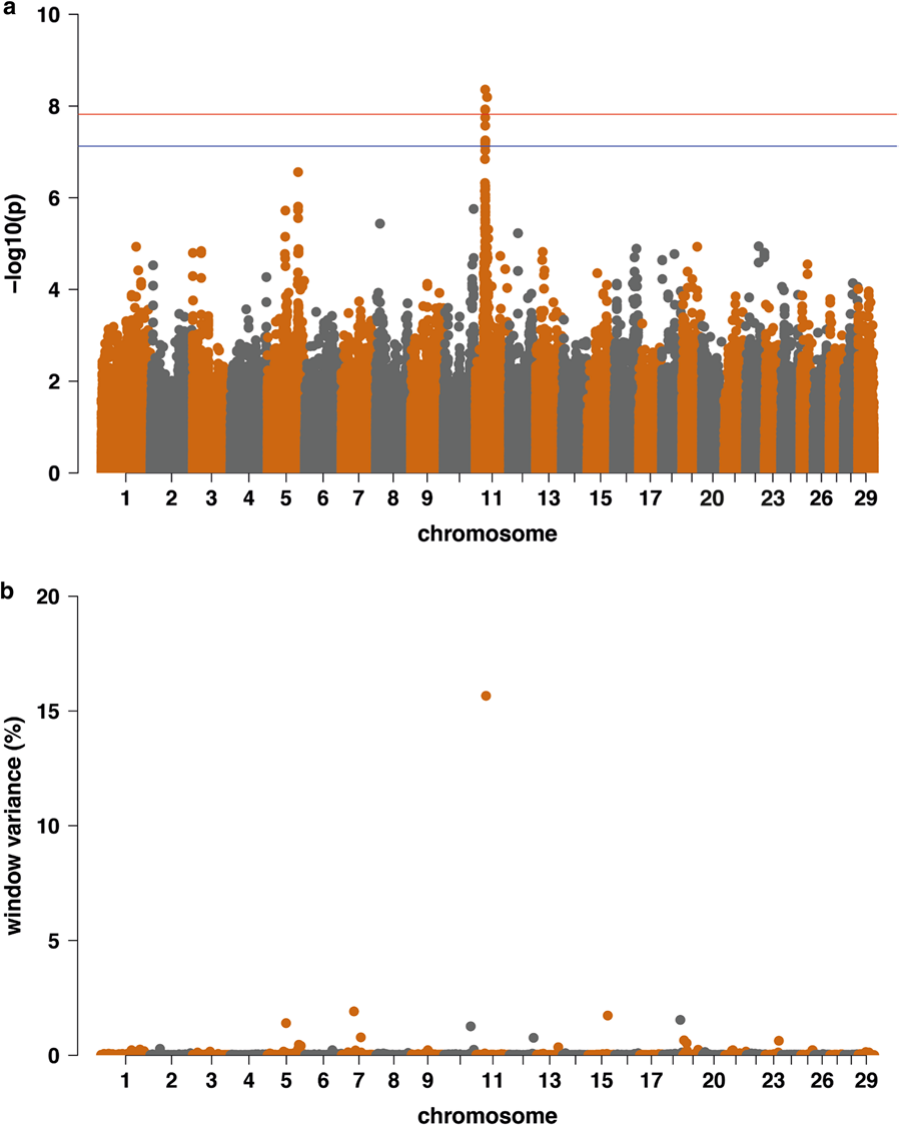
Manhattan plot of GWAS for the mbm trait. **a** For the single SNP regression, with Bonferroni corrected threshold level of 5% (blue line) and 1% (red line) and **b** for the window-based BayesB approach

Single SNP regression GWAS models showed 15 significantly associated SNPs at the Bonferroni corrected level of 5% for the mbm trait (Table 4). Four SNPs were significantly associated at the Bonferroni corrected level of 1% (Fig. 3a). Fourteen of these 15 SNPs defined a QTL on chromosome 11 between positions 31,022,855 and 31,337,157 bp (Table 4). An additional significantly associated SNP was identified in a different region on the same chromosome at position 37,136,773 bp. The most highly associated SNP was identified at position 31,004,983 bp. For the second trait analyzed (mbd), we did not observe any significant associations.

**Table 4:** Significantly associated markers from single SNP regression models for the mbm trait

Chromosome	Position	SNP	Alleles	Frequency	Variance	p-value
11	31,004,983	rs110112100	G/A	0.539	126.161	4.369 e−09
11	37,136,773	rs41579835	G/T	0.863	101.135	6.365 e−09
11	31,034,069	rs136576573	T/G	0.490	117.056	1.180 e−08
11	31,022,855	NA	A/G	0.490	117.346	1.206 e−08
11	31,037,875	rs42634817	G/A	0.490	114.435	1.771 e−08
11	31,049,877	rs135661502	C/T	0.581	112.918	2.680 e−08
11	31,329,763	rs43677285	C/T	0.633	100.127	5.626 e−08
11	31,330,363	rs43677273	G/T	0.633	100.127	5.626 e−08
11	31,332,310	rs43677261	G/A	0.633	100.127	5.626 e−08
11	31,336,575	rs43677231	A/C	0.365	99.800	5.931 e−08
11	31,337,157	rs43676629	A/G	0.365	99.800	5.931 e−08
11	31,338,042	rs136274250	C/T	0.635	99.800	5.931 e−08
11	31,060,572	rs135937618	C/A	0.581	106.172	6.378 e−08
11	31,044,741	rs137648582	A/G	0.431	103.469	7.291 e−08
11	31,070,514	rs133193362	A/C	0.419	105.105	7.383 e−08

Based on the ASR-UCD1.2/bosTau9 assembly

#### BayesB approach

We used a window size of 25 SNPs in the window-based BayesB approach. For the trait mbm, a significantly associated window that explained 15.66% of its genetic variance was identified (Fig. 3b) on chromosome 11 between positions 31,001,894 and 31,995,008 bp (Table 5). The values of p > 0 (proportion of models where this window was included, and therefore accounted for more than 0% genetic variance) and of p > Average (proportion of models where this window accounted for more than the amount of variance that would be explained if every window had the same effect) were both equal to 1. In the BayesB approach, the QTL reached a significance level of 1%, and thus, the significantly associated window on chromosome 11 overlapped with most of the significantly associated markers from single SNP regression. The other five possibly associated windows, each on a different chromosome, were detected below the significance threshold of 5% and explained smaller amounts of genetic variance (< 2%) (Table 5).

**Table 5:** Associated genome regions from the BayesB window approach for the mbm trait

Chr	Start position	End position	Number of SNPs	Proportion of explained genetic variance^a^	Cumulative proportion of varG^a^	p > 0	p > Average
11	31,001,894	31,995,008	345	15.66	15.66	1	1
7	39,005,627	39,999,299	266	1.91	17.58	0.942	0.416
15	62,002,607	62,994,714	242	1.73	19.31	0.942	0.404
18	56,015,521	56,987,121	344	1.54	20.85	0.987	0.363
5	59,005,181	59,981,866	173	1.40	22.25	0.850	0.291
10	85,003,025	85,988,287	327	1.26	23.51	0.972	0.351

*Chr* chromosome

p > 0 = proportion of models where this window was included, and therefore accounted for more than 0% genetic variance

p > Average = proportion of models where this window accounted for more than the amount of variance that would be explained if every window had the same effect

^a^In %

Subsequently, we inspected the gene content of the significantly associated QTL region on chromosome 11, and interestingly, we identified only two genes including their regulatory flanking regions in this segment, according to the NCBI Annotation Release 106: *LHCGR* and *FSHR*, which both represent obvious candidate genes for multiple births*.* The *LHCGR* gene (chr11:30,977,805–31,040,344) encodes the luteinizing hormone/choriogonadotropin receptor and the *FSHR* gene (chr11:31,255,649–32,450,537) encodes the follicle stimulating hormone receptor.

#### Haplotype analysis

We selected a 2-Mb-segment on chromosome 11 that starts at 30.5 Mb for haplotype association analyses based on the association results shown above. As discussed, this region encompasses two candidate genes including their regulatory regions. The 36 top-associated haplotypes according to their significance level from the association test are in Table 6. They encompass 19 to 37 SNPs and are located in the region between 31.00 and 31.07 Mb on chromosome 11. We selected one of the longest haplotypes (chr11:31,003,265–31,074,419) for further analysis, which included 37 SNPs and had a frequency of 0.275 in the entire genotype dataset (Table 6). An estimated additive effect of − 8.618 (± 1.188 standard error) was found for this haplotype, which indicates that it has a negative effect on the trait mbm.

**Table 6:** Top associated haplotypes from the regression analysis on chromosome 11 for the mbm trait

Start position	End position	Number of SNPs	Frequency	Effect	p-value
31,003,265	31,074,419	37	0.275	− 8.618 (1.188)	5.435 e−13
31,003,265	31,072,259	35	0.275	− 8.618 (1.188)	5.435 e−13
31,004,983	31,076,106	37	0.275	− 8.618 (1.188)	5.435 e−13
31,004,983	31,073,807	35	0.275	− 8.618 (1.188)	5.435 e−13
31,013,207	31,074,419	35	0.275	− 8.618 (1.188)	5.435 e−13
31,013,207	31,072,259	33	0.275	− 8.618 (1.188)	5.435 e−13
31,018,127	31,076,106	35	0.275	− 8.618 (1.188)	5.435 e−13
31,018,127	31,073,807	33	0.275	− 8.618 (1.188)	5.435 e−13
31,020,736	31,074,419	33	0.275	− 8.618 (1.188)	5.435 e−13
31,020,736	31,072,259	31	0.275	− 8.618 (1.188)	5.435 e−13
31,022,855	31,076,106	33	0.275	− 8.618 (1.188)	5.435 e−13
31,022,855	31,073,807	31	0.275	− 8.618 (1.188)	5.435 e−13
31,023,833	31,074,419	31	0.275	− 8.618 (1.188)	5.435 e−13
31,023,833	31,072,259	29	0.275	− 8.618 (1.188)	5.435 e−13
31,034,069	31,076,106	31	0.275	− 8.618 (1.188)	5.435 e−13
31,034,069	31,073,807	29	0.275	− 8.618 (1.188)	5.435 e−13
31,037,875	31,074,419	29	0.275	− 8.618 (1.188)	5.435 e−13
31,037,875	31,072,259	27	0.275	− 8.618 (1.188)	5.435 e−13
31,041,776	31,076,106	29	0.275	− 8.618 (1.188)	5.435 e−13
31,041,776	31,073,807	27	0.275	− 8.618 (1.188)	5.435 e−13
31,043,114	31,074,419	27	0.275	− 8.618 (1.188)	5.435 e−13
31,043,114	31,072,259	25	0.275	− 8.618 (1.188)	5.435 e−13
31,044,741	31,076,106	27	0.275	− 8.618 (1.188)	5.435 e−13
31,044,741	31,073,807	25	0.275	− 8.618 (1.188)	5.435 e−13
31,046,036	31,074,419	25	0.275	− 8.618 (1.188)	5.435 e−13
31,046,036	31,072,259	23	0.275	− 8.618 (1.188)	5.435 e−13
31,049,190	31,076,106	25	0.275	− 8.618 (1.188)	5.435 e−13
31,049,190	31,073,807	23	0.275	− 8.618 (1.188)	5.435 e−13
31,049,877	31,074,419	23	0.275	− 8.618 (1.188)	5.435 e−13
31,049,877	31,072,259	21	0.275	− 8.618 (1.188)	5.435 e−13
31,054,311	31,076,106	23	0.275	− 8.618 (1.188)	5.435 e−13
31,054,311	31,073,807	21	0.275	− 8.618 (1.188)	5.435 e−13
31,055,164	31,074,419	21	0.275	− 8.618 (1.188)	5.435 e−13
31,055,164	31,072,259	19	0.275	− 8.618 (1.188)	5.435 e−13
31,055,946	31,076,106	21	0.275	− 8.618 (1.188)	5.435 e−13
31,055,946	31,073,807	19	0.275	− 8.618 (1.188)	5.435 e−13

### Fine-mapping to the *LHCGR* locus

**Fig. 4:**
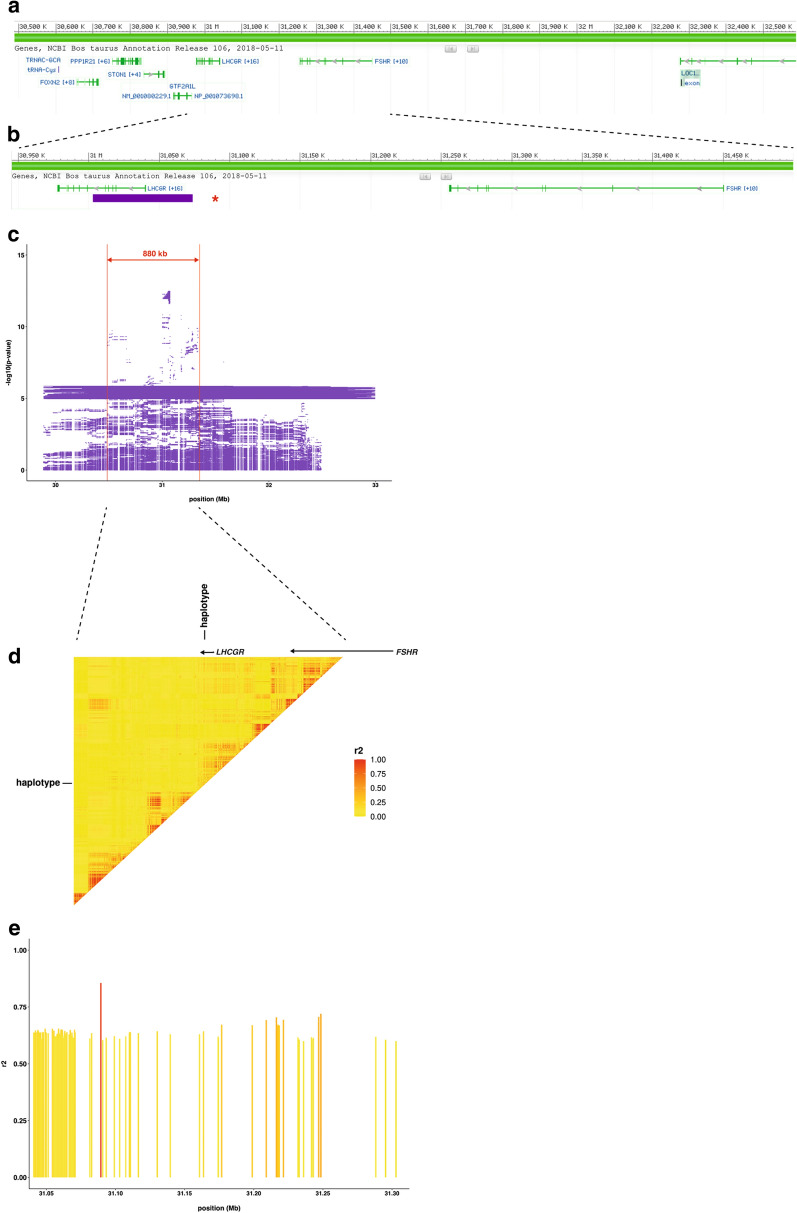
Graphical representation of the associated QTL region and linkage disequilibrium (LD) analysis results for the mbm trait. **a** Screenshot of the region on bovine chromosome 11 between 30.5 and 32.5 Mb from the NCBI Genome Browser including the localization of the genes in the region. **b** Screenshot of the region on bovine chromosome 11 between 30.95 and 31.5 Mb from the NCBI Genome Browser including the localization of the candidate genes *LHCGR* and *FSHR*. The purple bar shows the localization of the top-associated haplotype. The red star represents the variant with the highest LD score. **c** The resulting haplotypes from the haplotype regression analysis are shown with their localization. The 880-kb region with the top-associated haplotypes from 30.47 to 31.35 Mb used for subsequent fine-mapping is highlighted in red. **d** Heatmap showing the LD in the region between 30.47 and 31.35 Mb on bovine chromosome 11 highlighting the haplotype that was added as an additional variant. The localization and chromosomal orientation of *LHCGR* and *FSHR* are displayed (black arrows). **e** The variants in r^2^ ≥ 0.6 with the top-associated haplotype are highlighted and shown with their position on bovine chromosome 11

To unravel the most likely causal variant, we phased all genotyped samples for the chr11:31,003,265–31,074,419 haplotype using a routine imputation pipeline. Then, we merged the WGS data with the individual diplotypes for this haplotype and calculated the pairwise LD (r^2^) between the haplotype and all the detected variants in the VCF file for the 880-kb region of the top-associated haplotypes (Fig. 4c). All diplotype genotypes in the WGS samples were identical across all the 36 top-associated haplotypes (Table 6). Six variants located between 31.08 and 31.24 Mb on chromosome 11 showed r^2^ values ≥ 0.7 (Table 7), but none was in perfect LD (r^2^ = 1) with the haplotype (Fig. 4e). The variant chr11:31,089,325C>G had by far the highest r^2^ (0.856) (Fig. 4e), which as the other five variants, mapped to the intergenic region between the *LHCGR* and *FSHR* genes, close to the 5ʹ-region of *LHCGR* (Fig. 4b). None of these six variants are located in the top-associated haplotype between 31.00 and 31.07 Mb as described above (Table 7).

**Table 7:** Results of the linkage disequilibrium (LD) analysis for the mbm trait

Variant on chromosome 11^a^	Minor allele frequency	LD (r^2^)
31,089,325C>G	0.286	0.856
31,216,357C>T	0.334	0.705
31,246,990AGCC>-	0.326	0.707
31,248,462G>A	0.326	0.720
31,248,573C>T	0.333	0.706
31,248,580G>T	0.333	0.706

^a^ASR-UCD1.2/bosTau9 assembly

We performed an analysis of the sequence homology of the six variants identified in high linkage with the associated haplotype between 20 mammalian species using the UCSC genome browser to search for evidence of a putative causal role of these variants. Only one variant showed a homology score higher than zero, but its low value (0.071) indicates a low level of conservation (Table 8). The overall mean homology for the genomic region of the six variants was equal to 0.127.

**Table 8:** Results of the sequence homology analysis for all the variants with r^2^ ≥ 0.7 from linkage disequilibrium analysis

Variant on chromosome 11^a^	Homolog region for human genome^b^	r^2c^	Homology score^d^
31,246,990AGCC>-	2:48,957,377	0.707	0.071
31,089,325C>G	2:48,806,473	0.856	0
31,216,357C>T	2:48,930,243	0.705	0
31,248,462G>A	2:48,957,970	0.720	0
31,248,573C>T	2:48,957,971	0.706	0
31,248,580G>T	2:48,957,971	0.706	0

^a^Based on the cattle assembly ASR-UCD1.2/bosTau9

^b^Using the USCS genome browser and human assembly GRCh38/hg38

^c^From a previous linkage disequilibrium analysis with top-associated haplotype

^d^Sequence homology between 20 mammalian species using the UCSC genome browser, score range from 0 to 1

**Fig. 5:**
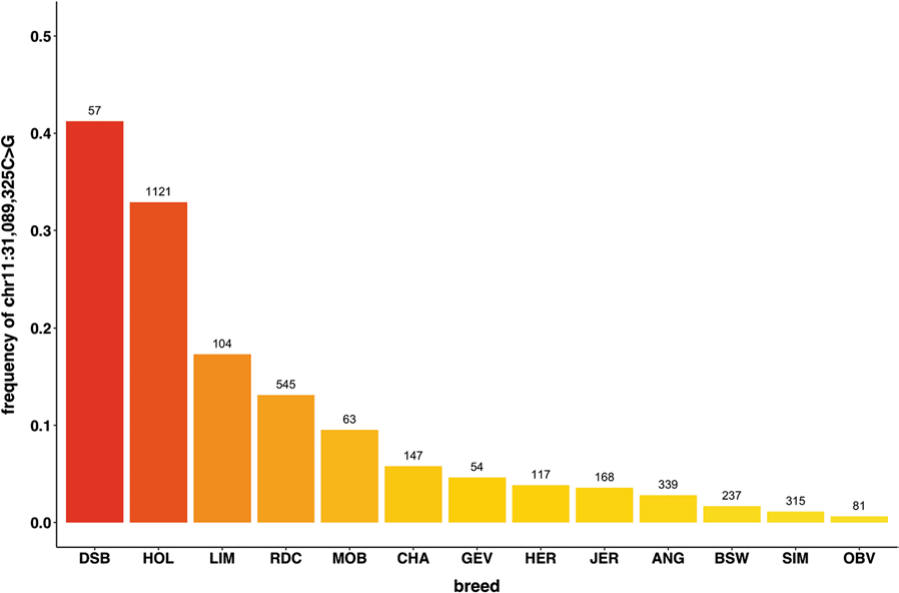
Frequencies among breeds for the top-linked variant. Calculated from the data of 1000 Bull Genomes Project for the chr11:31,089,235 C>G variant. Breed abbreviations: *ANG* Angus, *BSW* Brown Swiss, *CHA* Charolaise, *DSB* Deutsches Schwarzbuntes Niederungsrind, *GEV* Gelbvieh, *HER* Hereford, *HOL* Holstein, *JER* Jersey, *LIM* Limousine, *MOB* Montbéliarde, *OBV* Original Braunvieh, *RDC* Red Dairy cattle, *SIM* Simmental

Since the causative variants for the same trait can differ among breeds of the same species, we analyzed the frequencies of the variant on chromosome 11 in different breeds for all 4109 records in the vcf file provided through the 1000 Bull Genomes Project. The analysis was performed only for the variant in high LD (r^2^ ≥ 0.8) with the top-associated haplotype for all breeds that had at least 50 records. Interestingly, the frequency of this variant (chr11:31,089,325C>G) was highest for the Deutsches Schwarzbuntes Niederungsrind, the founder breed of modern Holstein cattle (Fig. 5), but frequencies higher than 10% were also found in Limousine and Red Dairy cattle. Obviously, this variant segregates in other breeds than Holstein, and also in related and unrelated breeds.

### Haplotype effects on routinely recorded fertility and birth traits

To evaluate a possible co-association between the most significantly associated chr11-haplotype for mbm, haplotype effects on routinely available fertility and birth traits were estimated. We found that only two specific traits, days to first service (DFS) and stillbirth maternal (SBM), showed a significant association at the 5% level (Table 9). The associated effects between the chr11:31,003,265–31,074,419 haplotype and these traits were positive. For other birth and fertility related traits, such as non-return rate cow, a suggestive association was observed at a significance level of 15%. The estimated effect for the chr11:31,003,265–31,074,419 haplotype on non-return rate in cows was negative. Taken together, these results suggest a clear effect of the identified haplotype on different female fertility traits.

**Table 9:** Effect of the top-associated chromosome 11 haplotype on other female fertility and birth traits

Trait group	Trait	Effect^a^	p-value^b^
Female fertility	Days to first service	0.664 (0.277)	0.017
	Interval between first and last insemination heifers	0.653 (0.495)	0.187
	Interval between first and last insemination cows	− 0.091 (0.257)	0.724
	Non-return rate heifers^c^	0.264 (0.350)	0.450
	Non-return rate cows^c^	− 0.436 (0.285)	0.125
Birth	Birth weight direct	− 0.210 (0.438)	0.632
	Birth weight maternal	− 0.687 (0.509)	0.177
	Calving ease direct	0.218 (0.199)	0.274
	Calving ease maternal	− 0.002 (0.298)	0.996
	Gestation length direct	− 0.071 (0.434)	0.870
	Gestation length maternal	− 0.580 (0.511)	0.256
	Stillbirth direct	1.020 (0.772)	0.186
	Stillbirth maternal	1.297 (0.524)	0.013

^a^Standard error of the effect in brackets

^b^Not corrected for multiple comparisons

^c^After 56 days

## Discussion

Decreasing female fertility is an acknowledged issue in high-performance dairy production and the occurrence of multiple births has long been an undesired trait. In this study, which is based on large-scale phenotyping and genotyping data, we have detected for the first time genetic factors that contribute to this trait. The estimated breeding values for maternal and direct multiple births were used as phenotypes in association studies. Two alternative GWAS approaches were applied and identified a major QTL on bovine chromosome 11 in a region that harbours two plausible candidate genes, *LHCGR* and *FSHR*, which directly affect the female reproduction cycle. We used the top-associated haplotype to identify variants in high LD with this region in the WGS data of hundreds of Holstein genomes. We found only one variant that was located in the 5ʹ-regulatory region of the *LHCGR* gene and represented a potential causal candidate.

The values of the estimated genetic parameters (variance components of direct and maternal effect for multiple birth) were similar to those in the literature [7, 15]. The development of a procedure to predict breeding values for binary traits, such as multiple birth, is not trivial because the phenotypic observations are not normally distributed. The use of linear mixed models that require normally distributed phenotypes can lead to acceptable rankings of animals according to estimated breeding values as shown by Negussie et al. [56] but the inferred results might not be valid and predictions do not have the same support as the discrete response variable. These challenges may help explain the extreme outlier values that we obtained here for both traits (mbd and mbm) and the smaller standard deviation of the estimated breeding values for mbm. The potential benefits of using a generalized linear mixed model or a threshold model in the context of this study are the subject of future research. Previous studies have already shown how to use generalized linear mixed models or threshold models for genetic evaluations in a general context as well as for multiple birth analysis [9, 30].

In the recent past, no clear genetic trend for the two studied traits (mbd and mbm) has been observed in the Swiss data in contrast to previous findings in Norwegian cattle [28] that showed a positive trend. Hence, it seems very likely that multiple births have not been under selection in the studied Swiss Holstein population. Thus, the prediction of breeding values could represent an important selection tool for reducing the occurrence of multiple births, which will hopefully lead to improved animal health and welfare. Availability of these results will allow to implement selection programs in the local breeding schemes.

In our dataset, we found only one clear QTL for the maternal trait, which underlines the essential role of the maternal component for the complex multiple birth trait. This QTL on chromosome 11 was detected by two GWAS approaches: single SNP regression and a window-based BayesB approach. Although single marker detection can be useful to detect many associations, only a small fraction of the genetic variance of quantitative traits can be significantly highlighted and identified with this approach [57–59]. The applied BayesB approach, used as a method that fits all the markers as random effects simultaneously, can account for most of the genetic variance [60–62] as well as a window-based approach that captures most of the variability at an associated trait locus [50]. The fact that the same QTL was detected by these two approaches provides strong evidence for the corresponding genomic region. The identified QTL explained 15.66% of the genetic variance of the trait. Furthermore, only one significantly associated SNP was detected by single SNP regression and was located downstream on the same chromosome, which indicates a single possibly false positive association signal. However, since mbm represents a classical polygenic trait, additional QTL of smaller effect might be detected with a larger sample size. The non-significantly associated segments on five other chromosomes observed in the window-based BayesB approach might represent suggestive QTL that need to be confirmed in the future. The theory of a polygenic trait is supported by previous studies, which revealed multiple QTL on various chromosomes [15, 31–38]. Regarding chromosome 11, only combined linkage-linkage disequilibrium analysis for North American Holstein sires revealed a QTL for twinning rate on the same chromosome but located in a different segment [31]. Those results were confirmed later in an additional American Holstein cattle population [34] but they were not validated in the USDA Meat Animal Research Center (USMARC) special herd that was selected for twinning rate [63]. Interestingly in the cattle QTL database, no QTL has been reported in the chromosome 11 region described here for the trait of interest [64]. Furthermore, this supports our assumption that this specific genome region, which is associated with multiple birth, has not been under selection in Holstein cattle, since the allele frequency has not changed significantly over time. In the literature, there is little evidence for a major QTL for multiple birth traits in cattle. Only two studies using data from North American Holstein and Norwegian cattle [32, 36] suggested a positional candidate gene with a possible major impact on the trait, i.e. *IGF1*. The region on chromosome 5 containing *IGF1* does not overlap with the suggestive QTL detected in our analysis. However, one can speculate that a larger and/or global dataset might improve such analyses and reveal additional QTL in Holstein and/or other cattle breeds.

The two genes, *LHCGR* and *FSHR*, which are located in the identified QTL region, are obvious candidate genes for multiple birth since they encode receptors of three essential hormones for female reproduction: luteinizing hormone (LH), choriogonadotropin, and follicle stimulating hormone (FSH). This is the first study that detects these genes as candidates for multiple birth in cattle.

Human chorionic gonadotropin is a hormone that is involved in the maternal recognition of pregnancy and is produced by trophoblast cells that surround the growing embryo, whereas LH is a hormone that is produced by gonadotropic cells in the anterior pituitary gland under the regulation of the gonadotropin-releasing hormone from the hypothalamus. Mutations in the human *LHCGR* gene that is expressed in the testis and ovary lead to disorders of the development of the male secondary sexual character, including familial male precocious puberty, also known as testotoxicosis, hypogonadotropic hypogonadism, Leydig cell adenoma with precocious puberty, and male pseudohermaphroditism with Leydig cell hypoplasia (OMIM 152790). In females, an acute increase in LH (LH peak) triggers the ovulation by initiating meiosis II in the oocyte at the point of ovulation and leads to follicle rupture and subsequent development of the corpus luteum [65, 66]. Interestingly, the concentrations of LH in the blood and plasma did not differ between selected and unselected bovine females for twin ovulations and dizygotic twins [67]. Therefore, we hypothesize that the identified variant in the 5ʹ-regulatory region of the bovine *LHCGR* gene might alter the expression of the encoded receptor in the ovary cell and thereby influence the ovulation rate explaining the effect on the studied multiple birth trait.

The FSH receptor is a transmembrane receptor that interacts with FSH and represents a G protein-coupled receptor that is expressed in the ovary, testis, and uterus. Mutations in the human *FSHR* gene cause ovarian dysgenesis type 1, and also the ovarian hyperstimulation syndrome (OMIM 136435). In the ovary, the FSH receptor is necessary for follicular development and is expressed on the granulosa cells and during the luteal phase in the secretory endometrium of the uterus. Although this gene can also represent a plausible functional candidate for multiple birth, the genetic findings presented here and the genomic localization of the identified potential causal variant provide stronger support for the *LHCGR* gene. However, an effect on *FSHR* expression cannot be fully ruled out. Interestingly, a study of the Flemish and Dutch human population provided some evidence that the same homologous region on chromosome 2 carrying the candidate genes *FSHR* and *LHCGR* had an effect on multiple birth [68]. In addition, a potentially functional variant in the 5ʹ untranslated region of *FSHR* has been reported in a single family [69], and two variants in the protein-coding area of *FSHR* have been detected in one woman with two twin pregnancies [70]. However, these findings were rejected by a study including 21 mothers with twins [71]. Recently, comprehensive GWAS studies for twinning rate in humans have become available, which revealed variants associated with the maternal trait in the *FSHB* and the *SMAD3* genes [72], and several QTL for the direct trait [73]. In general, the rate of monozygotic twins in humans is higher than in cattle, which might be a reason for the higher incidence of the direct effect. Our new findings could be used as a reference for other species, including humans.

Unfortunately, LD-based filtering of sequence variants within the critical segment did not filter out a single variant in perfect LD with the top-associated haplotype on chromosome 11. However, several non-coding variants showed high values of LD (r^2^ ≥ 0.7) that formed a 160-kb block. This was not unexpected due to the known relatively long-range LD pattern in cattle populations [74]. It has been shown that a single genomic region can harbor several QTL for a polygenic trait [75]. However, it is more likely that the identified segment that showed LD between the haplotype and several variants was due to LD between adjacent variants, while only one of them is the true causal variant. The effect on gene expression or regulation could also be influenced by multiple variants. The genomic localization suggested an effect of the variants on the regulation and expression of the *LHCGR* gene. Since the candidate variant (chr11:31,089,325C>G) segregates in various breeds at low frequency, it represents an old variation that most likely occurred before the formation of modern breeds. Ideally, a cross-breeding validation analysis should be performed. For most of the breeds analyzed here, neither phenotypes nor genotypes are available to evaluate a possible effect on multiple birth traits. In the data that was available for Brown Swiss and Simmental cattle, this variant showed low minor allele frequencies, which could be the reason why we were not able to confirm an effect in those populations (data not shown). Hence, we speculate that the pattern observed here is similar to that previously reported for the *DGAT1* p.Lys232Ala mutation that determines fat content in milk in Holstein and Brown Swiss, for which multiple variants showed a similar effect [76]. None of the six variants that had an LD value higher than 0.7 were located in the interval of the top-associated haplotype. They map downstream to this haplotype, but are still located in a block of elevated LD as shown in the presented heatmap (Fig. 4d). The top-associated haplotype identified is part of this LD block. However, to validate these findings, a larger dataset and other approaches such as differential gene expression analysis or targeted genome editing are necessary.

**Fig. 6:**
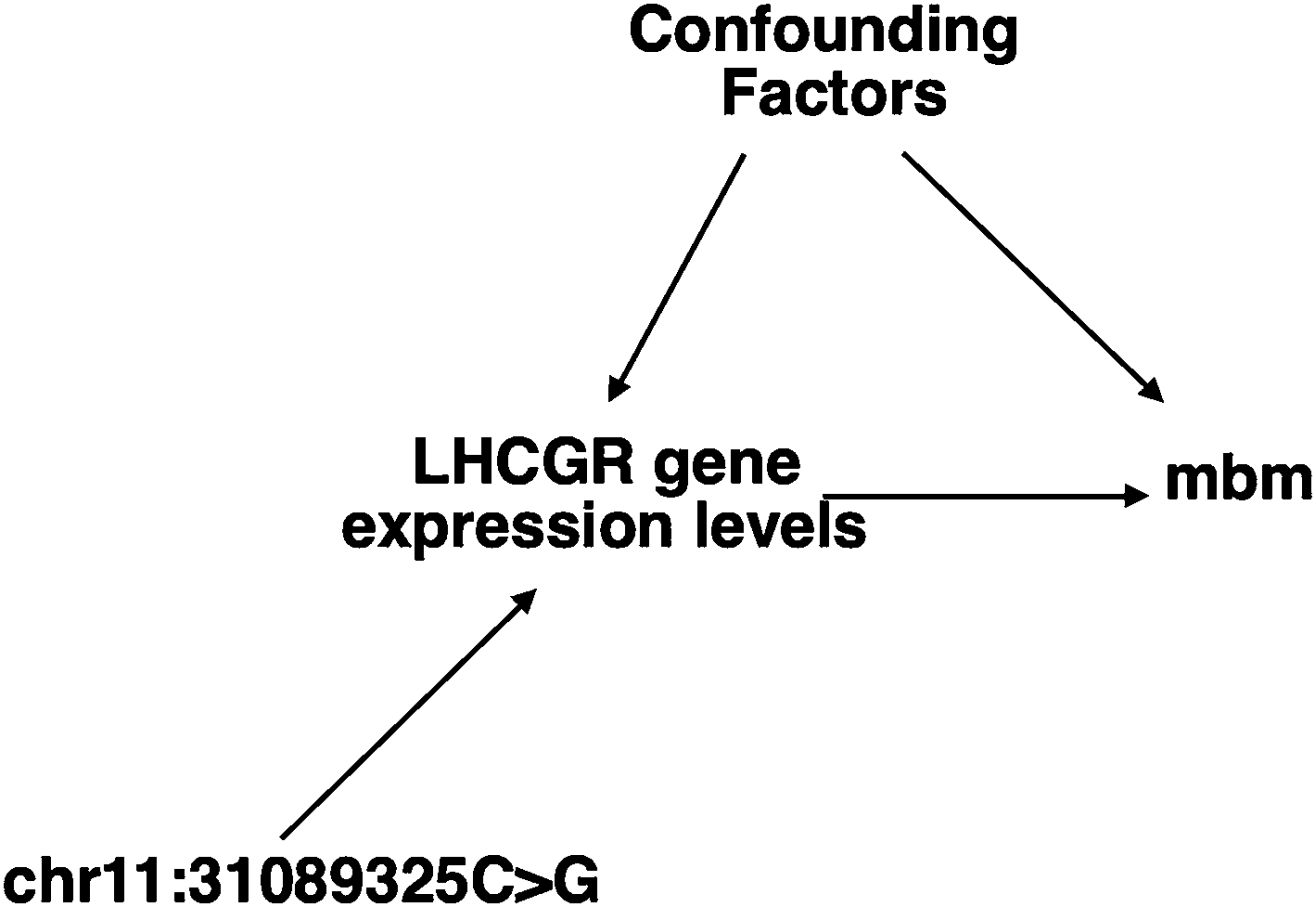
Direct acyclic graph denoting causality associations. The bovine chr11:31,089,325C>G genetic variant used as an instrumental variable in a Mendelian randomization analysis

Although the association reported for the SNPs found in this study strongly suggests a close connection to the *LHCGR* gene, a causal analysis based on Mendelian randomization (MR) as described in previous studies is required to make a definitive statement about the causality of the reported candidate location [77, 78]. In an MR analysis, the genetic variants (such as the chr11:31,089,325C>G variant detected here) are used as instrumental variables (IV) to disentangle the possibly confounded relationship between intermediate phenotypes such as *LHCGR* gene expression levels and our trait of interest (mbm). The three key assumptions for a genetic variant to qualify as an IV are shown in Fig. 6 and can be described as follows. First, the genetic variant (e.g. chr11:31,089,325C>G) has to be unrelated to typical confounding factors such as environmental factors, which is considered in our analysis, because the known confounding factors are included as fixed effects in our linear mixed effects model and therefore the influence of the confounding factors on the outcome trait (mbm) is taken into account. In the graph, the unrelatedness is encoded by the missing arrows between the nodes of the confounders and the genetic variant (Fig. 6). Second, the chr11:31,089,325C>G variant has to be associated with the exposure of the intermediate phenotype which in our case corresponds to the postulated effect on *LHCGR* gene expression. This means that the nodes of this genetic variant are connected to the intermediate phenotype. Third, conditional on confounders and exposure, the genetic variant and the outcome (mbm) are independent. Hence, if we know the levels of the exposure and the confounders, the genetic variant does not provide any additional information to the outcome trait (mbm). To test these three assumptions, we would need to have the observed values for the exposure of the intermediate phenotype which in our case are the expression levels of *LHCGR*, in addition to the genotypes of the genetic variant and the recorded events of our outcome trait (mbm). Unfortunately, the data for the intermediate phenotypes are not available in this study. The additional collection of intermediate phenotypes, such as the expression levels of *LHCGR* in order to be able to make a more definite statement about the causes behind the outcome trait of mbm, requires further research.

We compared the sequence homology of the six variants identified in high linkage with the associated haplotype between 20 mammalian species, which resulted in an homology score that is a measure of supporting evidence for a variant. The overall mean of the homology for the whole region for variants with an r^2^ value ≥ 0.7 was low, which is expected since our observed segment is an intergenic and non-coding region. Regarding the single variants, we did not observe any variants that showed high homology scores. Therefore, no further information can be obtained from this analysis regarding a potential causal role.

The detected associated effect of the identified haplotype on the studied trait mbm was negative. Hence, female haplotype carriers are expected to have a decreased incidence for multiple birth. Evaluation of the possible effects of the haplotype on other available birth and fertility traits revealed significant effects on days to first service and maternal stillbirth (Table 9). We found a positive significant effect on the female fertility trait, days to first service, which is associated with a larger interval between calving and first insemination, and a negative suggestive effect on non-return rate in cows, which is associated with a decreased insemination success. These two observations might provide supporting evidence for the influence of this genomic segment on female fertility, in general. In addition, we observed a positive effect on the birth trait ‘maternal stillbirth’, which results in a higher proportion of live birth events. Taken together, these observations support a most likely negative effect of the identified haplotype on female fertility including multiple births.

## Conclusions

Our aim was to undertake a comprehensive genetic analysis of multiple births in Swiss Holstein cattle. By analyzing large-scale genotype and phenotype data, we have detected, for the first time, a major QTL for maternal multiple birth on chromosome 11. One non-coding most likely regulatory variant located in the 5ʹ-region of the *LHCGR* gene was linked to the top-associated haplotype. This suggests that selecting against multiple births is possible, but a putative negative effect on other female fertility traits needs to be considered. These findings improve our understanding of the genetic architecture that underlies multiple birth in mammals and female fertility, in general, but further studies are needed to strengthen the evidence for a causal relationship between the detected *LHCGR* locus and the phenotype.

## Data Availability

The sequence data for all animals obtained from the 1000 Bull Genomes Project is available at the EVA (www.ebi.ac.uk/eva/). The SNP data analyzed during the current study are not publicly available but are available from the corresponding author on reasonable request.
